# Process of mercury accumulation in urban strip river artificial wetland ecosystems: a case study of Changchun, a typical industrial city in Northeast China

**DOI:** 10.3389/fpls.2024.1392904

**Published:** 2024-05-03

**Authors:** Jing Zong, Hongjie Zhang, Xuemei Li, Xinyu Bai, Yufei Hu, Dan Cui, Zhaojun Wang, Gang Zhang

**Affiliations:** ^1^ School of Environment, Northeast Normal University, Changchun, China; ^2^ Jilin Province Expressway Group Operating Development Co., Ltd, Changchun, China; ^3^ College of Languages and Cultures of Ningde Normal University, Ningde, China; ^4^ State Environmental Protection Key Laboratory of Wetland Ecology and Vegetation Restoration, Changchun, China; ^5^ Key Laboratory of Vegetation Ecology, Ministry of Education, School of Life Sciences, Northeast Normal University, Changchun, China; ^6^ Institute of Grassland Science, School of Life Sciences, Northeast Normal University, Changchun, China

**Keywords:** urban constructed wetlands, mercury, water, sediment, botany, bioconcentration factors

## Abstract

Mercury (Hg), as a global pollutant, is persistent, migratory, insidious, highly biotoxic and highly enriched, and is widely distributed in the atmosphere, hydrosphere, biosphere and lithosphere. Wetland ecosystems, as active mercury reservoirs, have become the most important sources and sinks of heavy metal mercury. Distinguished from natural wetlands, artificial wetlands located in urban sections of rivers face problems such as diverse urban pollution sources and complex spatial and temporal changes. Therefore, in this study, five intermittently distributed artificial wetlands were selected from the upstream to the downstream of the Changchun section of the Yitong River, a tributary of the Songhua River basin in the old industrial base of Northeast China. The mercury levels in the water bodies, sediments and plants of the artificial wetlands were collected and tested in four quarters from April 2023 to analyse the spatial and temporal distribution characteristics of total mercury. The results showed that the mercury levels in the water bodies, sediments and plants of the five wetlands showed a fluctuating trend with the river flow direction and had certain spatial and temporal distribution characteristics. This phenomenon was attributed to the sinking of external mercury pollution sources. In general, the wetland ecosystems showed a decreasing trend in the total Hg output of the downstream watershed. This may be due to the retention of particulate matter by aquatic plants in artificial wetlands to regular salvage of dead aquatic plants. At the same time urbanization and industrialization affect mercury levels in aquatic environments, so the risk of residential exposure needs to be looked at.

## 1 Introduction

Mercury is a global migratory heavy metal pollutant characterized with strong physiological toxicity, biological enrichment, and migration abilities (Ullrich et al., 2001; Obrist et al., 2017). At room temperature, mercury is a volatile silvery white liquid with low melting and boiling points. The use and storage of mercury began to be controlled as the dangers of mercury became better understood. According to the previous research results, after the gradual decrease of mercury emissions, it was thought that mercury pollution seemed to be under control within a certain scope and degree. This was mainly due to the significant decreases of mercury content levels in organisms in some previously severely polluted areas (Meade et al., 2004). However, in the early 1990s, new studies found that the mercury content levels in fish remained high in some lakes which were originally considered to be located far away from mercury pollution sources (Meili, 1991). This has led to the recognition of mercury as a pollutant which has the ability to migrate on a global scale (Driscoll et al., 2013). Gaseous elemental mercury has poor water solubility and cannot easily settle. Unfortunately, the form of mercury in atmospheric mercury is mainly gaseous elemental mercury, which accounts for more than 90%. Therefore, atmospheric mercury can remain in the atmosphere for long periods of time (approximately 0.5 to 2 years) and migrate with the air currents over long distances (Li et al., 2022).

High levels of mercury in organisms can lead to mercury poisoning. In the 1950s, the Minamata disease incident which occurred in Japan’s Kumamoto Prefecture confirmed that mercury can accumulate and amplify in the ecosystem along the food chain. Damages to human and animal systems and organs can result, causing death in severe cases. The aforementioned incident also made people fully aware of the potential danger of mercury accumulation.

The content levels of anthropogenic atmospheric mercury in China approximately range from 500 to 700 tons/year, among which the annual average release of coal burning and smelting accounts for 80% of the total release (Zhu et al., 2012). From the beginning of the industrial revolution to 2003, the content levels of mercury in global water bodies, atmosphere, and soil have increased by approximately three times, particularly in the vicinity of light and heavy industrial areas. The term “chemical time bomb” has been used to vividly describe the delayed effects of mercury pollution on the environment and the subsequent harm which will be cause (Futsaeter and Wilson, 2013).

The main sources of mercury can be divided into anthropogenic and natural sources. Anthropogenic sources mainly include production and living activities, such as traffic, smelting, waste incineration, and fuel combustion. Natural sources mainly include such natural disasters such as volcanic eruptions and forest fires (Luo et al., 2016; Liu et al., 2021). The sources of mercury pollution in water bodies include excessive discharges of industrial wastewater containing heavy metal mercury, rainwater scouring, and atmospheric subsidence, as well as mercury and its compounds within soil entering surface water or groundwater (Futsaeter and Wilson, 2013). Excessive discharge of industrial wastewater is the main source of mercury pollution in China’s water bodies (Zhang et al., 2021).

In China, there are many industrial industries which produce and discharge mercury, and the discharge of industrial wastewater is the main cause of mercury pollution in water bodies. In addition, the sources of mercury in water are atmospheric deposition, precipitation runoff, and soil mercury leaching into the wetland systems with water flow (Lusilao-Makiese et al., 2016).

Particle matter is one of the carriers of most heavy metals. Since particle matter has the functions of adsorption, deposition, and migration, most heavy metals in water are combined with the surfaces of suspended particulate matter (Henry et al., 2008). According to suspended particle adsorption processes, such as the attachment of toxic heavy metals on its surfaces, the attachment of the majority of toxic heavy metal which accumulates in suspended particulate matter is through the subsidence processes in water bodies at the bottom of the sediment layers. Therefore, in order to reduce the content levels of heavy metals in water bodies and reduce the toxicity of toxic heavy metals in aquatic organisms, these processes need to be clearly understood (Ankley et al., 1996).

As major reservoirs for heavy metals in water bodies, river sediment is mainly composed of various minerals, such as clay, sediment, and organic matter with large specific surface areas (Wang et al., 2020). Sewage discharge, atmospheric deposition, rainwater erosion, and wind erosion are known to be the main sources of mercury in river sediment (Randall and Chattopadhyay, 2013). There are at least three ways by which mercury can be released from sediment. The first is that the mercury in water bodies will migrate to the surface of suspended particles and colloids through diffusion and then become adsorbed and settled. The second the direct release of mercury into the aqueous phase through dissolution. The third way that mercury is released occurs when the element mercury is converted into nitric acid and escapes into the atmosphere in a gaseous state (Wang et al., 2010). In constructed wetlands with slow water flow, the sediment re-transport processes tend to be very weak, and the sediment layers polluted by mercury, namely the bottom mud layers, are relatively stable. The mercury deposited in sediment can cause potential pollution since it is maintained in water bodies for long periods of time. When the water environments are suitable, insoluble mercury can be changed into soluble mercury, and this process is repeatedly released from the bottom mud layers into the water above (Randall and Chattopadhyay, 2013). The temperature and pH values are the main factors affecting the adsorption and release behaviors of Hg from sediment. The adsorption capacity of sediment to heavy metals will increase with the increases in pH values (Zhong and Wang, 2008). Therefore, the organic matter content and pH values of sediment in constructed wetlands are important influencing factors of the total mercury content in the sediment layers (Hsu-Kim et al., 2013).

Vegetation can absorb mercury through stomata and absorb atmospheric mercury from dry and wet deposition on leaf surfaces. The main routes of heavy metal mercury into plants include the following: (1) Atmospheric mercury enters through stomatal absorption in plants (Laacouri et al., 2013); (2) Soluble mercury within soil enters through transpiration processes in the underground tissues of plants; (3) Atmospheric mercury enters via leaf surface adsorption (Olson et al., 2019).

However, since almost all of the mercury content in mosses originates from the atmosphere, it has been found that different types of plants absorb mercury from various environments in different ways, degrees, and forms. This characteristic makes mosses one of the indicator plants for regional mercury deposition in the early stages (Li et al., 2023). Generally speaking, atmospheric mercury is the main source of the majority of the heavy metal mercury in plants, while the absorption of mercury by plants from soil is very limited (Schwesig and Krebs, 2003).

Mercury can be absorbed into plant tissues through migration, transformation, and redistribution. In addition, plants absorb mercury in sediment layers through their root systems, and the mercury entering plants is mainly accumulated in the underground tissues of the plants (Liu et al., 2003). However, the mercury content in the above-ground plant tissues accounts for only one hundred and ninety-eighth of the mercury content in the underground tissues of the plants. The mercury content in the underground tissues of plants can be approximately three times that of the mercury content in the sediment layers around the living environments of the plant roots (Vane et al., 2009; Guo et al., 2020). A large number of studies have shown that the plant types which absorb heavy metal mercury mainly include woody plants, herbs, and the above-ground parts of shrubs via the absorption of mercury from the atmosphere. The mercury in plant roots is mainly derived from the absorption of mercury from the soil (Ericksen and Gustin, 2004; Millhollen et al., 2006). In addition, plant roots can also secrete organic compounds and have better coordination ability for heavy metals. In summary, the interactions between roots and sediment are very complex, and the amount of heavy metal uptake by plants is dependent on the availability of heavy metals in sediment, which is limited by various sediment and plant factors (Weis and Weis, 2004).

One of the most important components of mercury in the global cycle is the various environmental behaviors of mercury in wetland ecosystems (Driscoll et al., 1994). As active reservoirs of mercury, wetland ecosystems are extremely important sources and sink areas for heavy metal mercury (Bachand et al., 2019). Natural wetlands play important roles in maintaining the health of ecosystems. Therefore, the value of natural wetlands, and their impacts on human, animal, and plant living environments, can no longer be ignored.

However, during the past 100 years, 64% to 71% of the natural wetlands on the Earth have disappeared (Davidson, 2014). In order to simulate the working principles and operating environments of natural wetlands by learning the relevant operating principles of natural wetlands, constructed wetlands have been designed to amplify the various functions of natural wetlands, maximize and optimize the functions of natural wetlands, and address various types of combined urban ecological environmental problems.

The main purposes of constructing artificial wetlands involve the treatment of sewage and wastewater with good controllability. Constructed wetlands often depend on the adsorption effects of filler, plant absorption, and the synergy of microbial metabolism in order to remove contaminants. The pollutants generally accumulate in organic matter, such as toxic heavy metals, but also have certain nutrients for common absorption purification effects. Therefore, they are fundamentally related to substrates, plants, and the growth of microbial composition (Zhang and Wang, 2018). The release of mercury from soil parent material is the main source of mercury in soil. However, that only applies to some areas with typical geological conditions. Generally speaking, the main sources of mercury in regional wetlands are atmospheric dry and wet deposition and runoff input (Mitsch et al., 2005). For example, for wetland ecosystems far from industrial activity areas, one of the key sources of mercury input is atmospheric dry and wet deposition.

In recent years, mercury pollution has spread across the globe. According to the latest statistics, more than 1×10^4^ t of mercury is excavated for industrial production in the world every year. The annual mercury discharged into the natural environment is estimated to be 5 × 10^3^ t. Even the Arctic region, where few people visit, has mercury pollution problems (Li et al., 2019a). The provinces of Jilin, Guizhou, Chongqing, Shaanxi, and Liaoning are considered to be the main concentration areas of mercury pollution in Chin (Kim et al., 2016).

Currently, the research regarding the ability of different plants to remove mercury is still the focus of international researchers in constructed wetland areas. The research results have shown that the majority of wetland plants can absorb the heavy metal mercury in water to a certain extent, and the constructed wetlands where such plants are planted have displayed the ability to efficiently remove mercury in water (Wang et al., 2013). At this time, the biogeochemical cycle of mercury is basically the focus of Chinese research in this field. The study of Hg content in wetland sediment, soil, vegetation, and benthic organisms, and the spatial and temporal distribution of mercury in lakes, reservoirs, and bays have been the main focus areas (Defne and Ganju, 2015; Wang et al., 2021). However, mercury interference from natural wetlands, point source pollution, and non-point source pollution in areas with severe mercury pollution remain the primary focus of mercury research in wetlands in both China and internationally (Zhao et al., 2019).

In this study, we analyzed the spatial and temporal distribution patterns of mercury in artificial wetlands by field sampling of artificial wetlands in the Changchun section of the Yitong River, further clarified the geochemical cycling of mercury in urban artificial wetlands, the impacts of urban artificial wetlands on the downstream watersheds, and the importance of wetland plants in the purification of pollutants in artificial wetlands, and clarified the degree of disturbance of wetland ecosystems by human activities and the wetland’s response mechanism, from the point of view of controlling potential Hg exposure risk level, with a view to providing suggestions for the construction of artificial wetlands.

## 2 Materials and methods

### 2.1 Overview of the study area

The Songhua River is one of the seven major rivers in China. It runs through Jilin and Heilongjiang Provinces, and runs four provinces: Inner Mongolia, Jilin, Liaoning, and Heilongjiang Provinces, with an area of 557,200 km^2^ and an annual runoff of 76.2 billion m^3^. Yitong River, the second tributary of the second Songhua River, is the source river of Changchun.

At an altitude of approximately 250 to 350 m, Changchun is located in the hinterland of the plain region of northeastern China. Its terrain is open and flat, and the precipitation decreases from east to west. Changchun is located in a transition zone from a humid zone to sub-arid zone, with the air temperature increasing from east to west (Wang et al., 2023).

**Figure 1:**
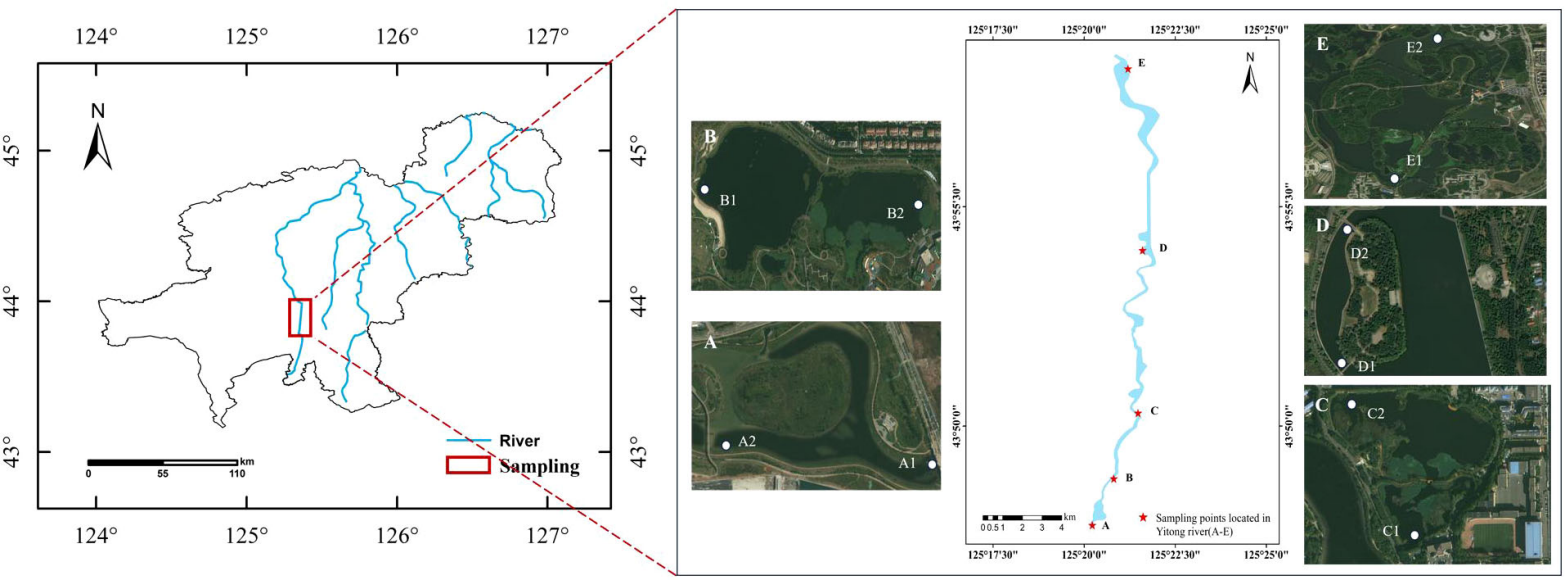
Geographical location of the study area.

There are also many types of wetlands in Changchun, which are rich in wetland resources. These include lake wetlands, swamp wetlands, river wetlands, and constructed wetlands. The geographical location of the study wetland is shown in **Figure 1**. As one of the main components of surface water resources, river wetlands are also important parts of the Changchun wetland region.

### 2.2 Sampling sites

In this study, five wetlands were selected from south to north along the Yitong River in the main urban area of Changchun (**Table 1**): A. South Fifth Ring Wetland; B. Rival Wetland; C. Beihai Wetland; D. Donglai South Street Wetland; and E. Bayhood Wetland. The water source of the selected wetlands were mainly river water, accompanied by a small amount of domestic sewage and water discharged by sewage plants, and their operation cycles were about 8-10 years. The water inlet and outlet of each wetland were set with sampling points denoted as A1-E2. The water sample points were recorded as W-A-1 to W-E-2; plant sample points were denoted as P-A-1 to P-E-2; and the sediment sample points were referred to as S-A-1 to S-E-2.

**Table 1:** Description of the geographical locations of the sampling points.

Sample number	Longitude and latitude of the sample plot	Sample name	Sample location	Sample description
A	125.346717,43.788495	South Fifth Ring Wetland	South of the Changchun Ring Expressway	This was the Yitong River inlet, located far away from residential areas and natural wetlands. The water is less mobile. The plants around the wetland were well preserved.
B	125.356089,43.816068	Rival Wetland	Rival Wetland Park	This sampling site was located in the southern section of Rival Wetland Park, with few residential areas, pedestrians, and vehicles nearby. The water surface was completely thawed, and the water flowed slowly, basically presenting a static state.
C	125.366403,43.84541	Beihai Wetland	Beihai Wetland Park	This sampling site was located in the northern section of Beihai Wetland Park, with dense residential areas nearby. The ice on the water was thick, with shovels unable to break through. Only a small part of the shore area was in a state of thaw, and the water mobility was poor.
D	125.366245,43.905138	Donglai South Street Wetland	Donglai South Street	This sampling site was located to the eastern section of Donglai South Street, with dense residential areas nearby. The water flow is evident.
E	125.370197,43.983103	Bayhood Wetland	Bayhood Wetland Park	This sampling site was located in the western section of the Bayhood Wetland Park, with few residential areas nearby and the largest wetland area. The water flow is slower.

### 2.3 Sample collection

In this study, water, sediment and plant samples were collected at different locations in four seasons starting from April 2023, with three replicates collected at each sampling site, for a cumulative total of 120 water samples, 120 sediment samples and 120 plant samples collected throughout the year. Sediment samples were collected near plant roots. Plant samples focused on plants near the water sample collection site.

#### 2.3.1 Water sampling

According to the “Technical Guidelines for River Sampling” HJ/T 52-1999, sampling points were set up for the water body of the artificial wetland, and samples of the water body were collected at each sampling point of the artificial wetland with a sampler. In order to prevent the disturbance of the water body during the sampling process from affecting the experimental results, the samples were taken in the upstream direction of the sampling boat to ensure the accuracy and reliability of the sampling. The collected water samples were stored in clean 200 ml borosilicate glass bottles, soaked in 30% nitric acid for 24 h, and then put into a 500°C constant temperature muffle furnace for ultra-purification for 30 min, and then cooled down and put on a double-layer plastic bag for use. Unfiltered water samples were collected from each sampling point and put into borosilicate glass bottles with a capacity of 200 ml, and a small amount of water was taken to clean the sample bottles three times before sampling. After the water samples were collected, 0.4% of 12 mol/L HCl solution was added immediately, and then the bottles were capped, packed in double plastic bags and labeled for transportation back to the laboratory for testing.

#### 2.3.2 Sediment sampling

A lake sediment sampler (SWB-1 portable) was used to collect surface sediment samples, and after the sediment samples were collected, the interface water was extracted with a siphon tube, which should not be completely pumped out in order to ensure that the sediments were kept in an anoxic state. As soon as possible after the extraction of interface water, the sediment samples were put into double-layer self-sealing bags under nitrogen-filled anaerobic conditions and brought back to the laboratory.

#### 2.3.3 Plant sampling

At each sampling point, collect four plants in good growing condition with approximately the same growth and height, and take samples from every other plant, keep the integrity of the plant when collecting, try to keep the root system intact, and do not damage the root hair part. Each sampling point was encapsulated in a polyethylene plastic bag, the sample label was written, and the sampling point and plant sample number were accurately recorded in the log book and brought back to the laboratory.

#### 2.3.4 Analysis of the samples

Collected water samples were transported back to the laboratory and stored in a refrigerator at a low temperature of 0 ~ 4°C. If the total mercury in the collected water samples could not be determined quickly, the water samples were frozen under freezing conditions until the total mercury content could be determined. After removing stones and dead leaves, the collected sediment samples were dried in a vacuum freeze dryer (- 80°C), then ground using a mortar and pestle and passed through a 100 mesh nylon sieve, and the ground sediment samples were stored in self-sealing bags for determination. When the plant samples were brought back to the laboratory, they were first rinsed with tap water to remove surface soil and dirt, and then rinsed three times with deionized water. The cleaned plants were dried naturally, and the roots, stems and leaves of four plants from each sampling point were mixed evenly and cut into pieces and put into a porcelain crucible, dried with a vacuum freeze-dryer (- 80°C), then ground with a mortar and pestle, passed through a 100-mesh nylon sieve, and stored in a bag for spare use.

A Lumex RA-915+ Zeeman effect mercury analyzer was used to determine the content levels, with an accuracy of 0.1 ng/g. Liquid samples were analyzed using a liquid mercury reduction unit, RP-92-, by cold vapor atomic absorption spectrometry (CVDAS) with a detection limit of 0.5 ng/L. The correlation coefficient of the standard curve of the aqueous samples was R^2^ of 0.9996. The sediment samples were determined using a Russian LUMEXRA-915 + coupled UMA solid-liquid mercury analysis unit with a detection limit of 0.5ug/kg. The quality control (QC) procedure was based on the calibration of the XRF instrument with reference to the SRM (Standard Reference Material) of the U.S.A. The correlation coefficient of the standard curve was 0.9996. The correlation coefficient of the standard curve for sediment samples is 0.9999. The concentration of plant samples was determined by thermal analysis using the PYRO-915+ accessory with a detection limit of 2ug/Kg. The correlation coefficient of the standard curve for plant samples is 0.9994.

### 2.4 Method for calculating the plant mercury enrichment coefficient

The biological enrichment coefficients represent the ability of organisms to enrich heavy metals from the soil, and refers to the ratio of the concentration of a certain heavy metal element in plant tissues to the concentration of the same heavy metal element in the soil. The higher the enrichment coefficient was, the stronger the enrichment ability would be. The calculation formula was as follows:


(1) $$BF=C_{P}/C_{S}$$

Where Cp represents the concentration level of mercury in the plant body; and Cs indicates the concentration of mercury in the root soil of the plant.

### 2.5 Statistical data method

In this study, SPSS software was used to statistically analyze the experimental data. After verifying the non-normal distribution of the data distribution (Shapiro-Wilks test (0.05) and unequal variances (Bartlett’s test (0.05), the non-parametric Kruskal-Wallis test was applied to compare the levels of Hg concentrations in the water column, sediments, and plants. Pearson’s correlation index (r) was applied to highlight possible interactions between Hg uptake in plants and the ratio of Hg concentrations in water bodies and sediments. Microsoft Excel was used for the preliminary analysis of the data and origin was used for the graphic plotting.

## 3 Experimental results

### 3.1 Mercury content levels in the water bodies

**Figure 2:**
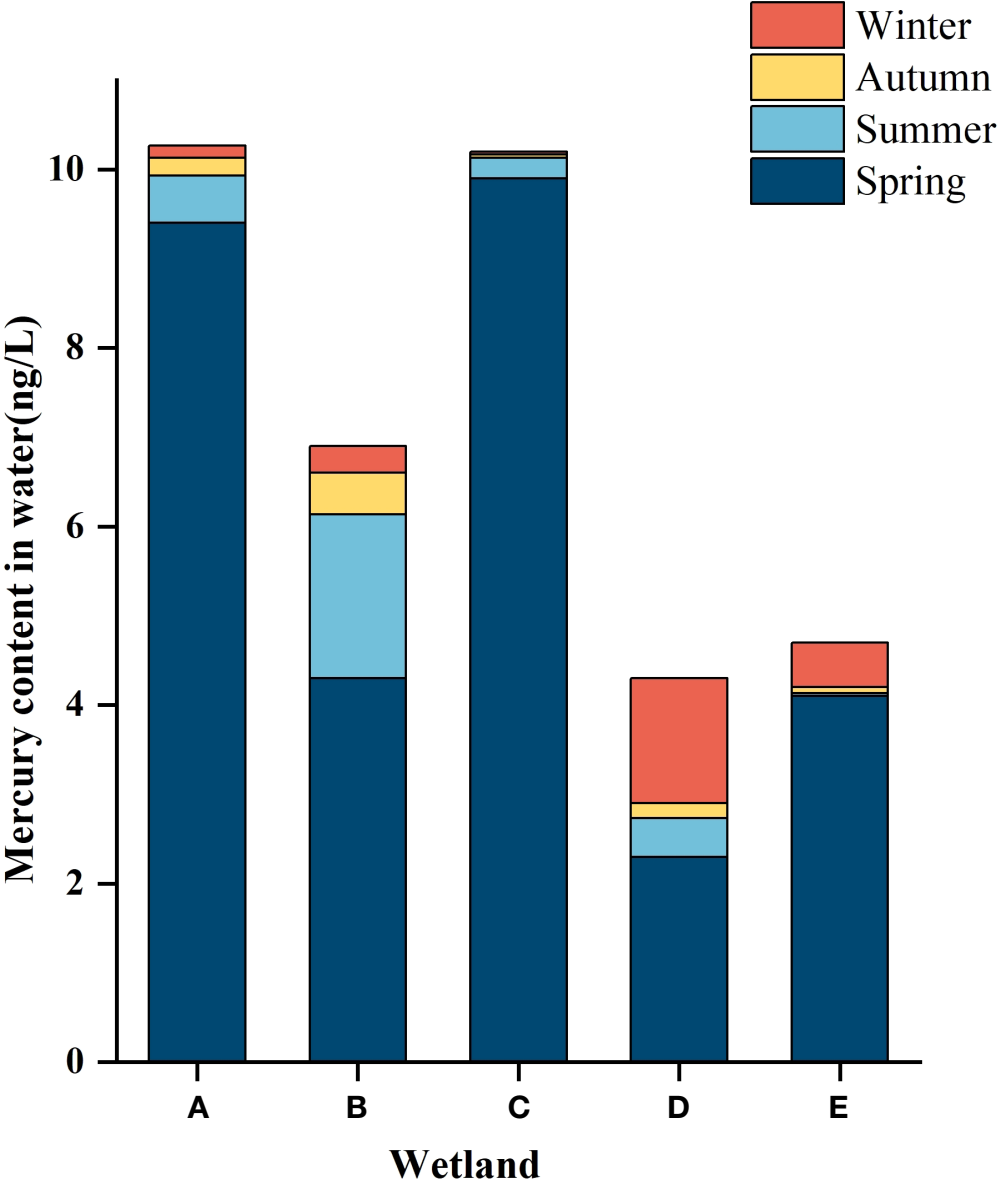
Hg content in the water of different constructed wetlands. **(A)** South Fifth Ring Wetland; **(B)** Rival Wetland; **(C)** Beihai Wetland; **(D)** Donglai South Street Wetland; **(E)** Bayhood Wetland.

As described in **Tables 2** and **3**, there was no significant difference in the mercury content in the water bodies of different wetlands in the same season, and the total mercury mass concentration in the water bodies of the five wetlands ranged from 0.033 ± 0.008ng/L to 9.9 ± 0.2ng/L, which was lower than the standard limit of mercury concentration in surface water (100ng/L) as stipulated in the Chinese environmental quality standard (GB3838-2002). As can be seen from **Figure 2**, spatially, the distribution of mercury in the water in the five wetlands showed a decreasing trend along the river flow direction, with an upward trend to the middle reaches of the river, followed by a decrease in the total mercury mass concentration to the lower reaches of the river.

**Table 2:** Description and statistics of the mercury content in the water bodies of the constructed wetland systems (unit: ng/L).

Wetland	Mercury content in Water(ng/L)			
	Spring	Summer	Autumn	Winter
South Fifth Ring Wetland	9.4 ± 0.2	0.6 ± 0.436	0.333 ± 0.153	0.2 ± 0.1
Rival Wetland	4.3 ± 0.5	1.833 ± 0.208	0.467 ± 0.289	0.3 ± 0.065
Beihai Wetland	9.9 ± 0.2	0.433 ± 0.216	0.167 ± 0.153	0.033 ± 0.008
Donglai South Street Wetland	2.3 ± 0.265	0.433 ± 0.115	0.167 ± 0.058	1.4 ± 0.173
Bayhood Wetland	4.1 ± 0.1	0.233 ± 0.208	0.067 ± 0.058	0.567 ± 0.443

**Table 3:** Seasonal differences in mercury levels in water bodies of different wetlands are significant.

	Spring	Summer	Autumn	Winter	P
South Fifth Ring Wetland	4.3(3.45,6.75)	0.90(0.70,1.45)	0.30(0.25,0.40)	0.20(0.15,0.75)	0.401
Rival Wetland	9.4(5.80,9.65)	0.50(0.30,0.65)	0.20(0.15,0.20)	0.30(0.20,0.95)	0.049
Beihai Wetland	9.60(6.80,9.85)	0.10(0.10,0.20)	0.30(0.20,0.30)	0.30(0.20,0.80)	0.087
Donglai South Street Wetland	4.80(4.50,7.25)	0.90(0.55,1.25)	0.20(0.20,0.25)	0.20(0.15,0.25)	0.028
Bayhood Wetland	3.80(2.95,3.95)	0.40(0.35,1.15)	0.10(0.10,0.45)	0.60(0.35,0.95)	0.047
P	0.331	0.182	0.614	0.924	

**Figure 3:**
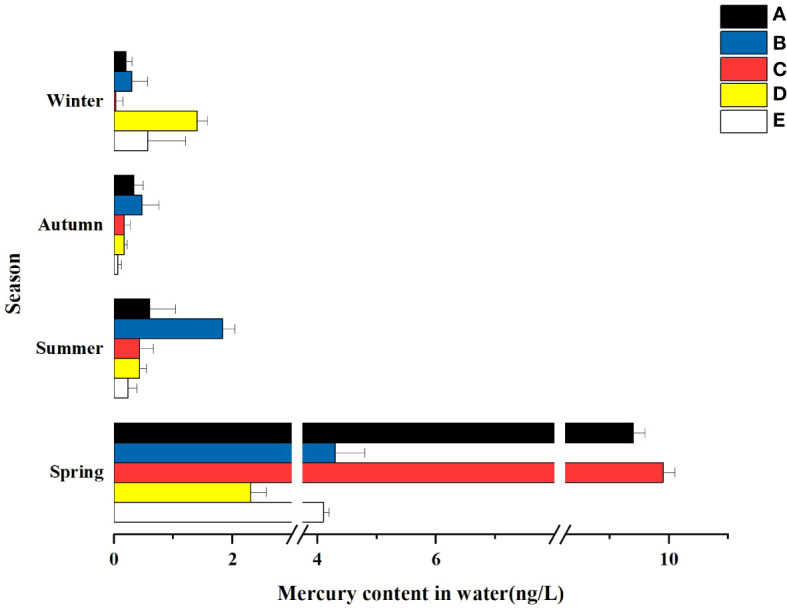
Hg content in the water of different constructed wetlands. **(A)** South Fifth Ring Wetland; **(B)** Rival Wetland; **(C)** Beihai Wetland; **(D)** Donglai South Street Wetland; **(E)** Bayhood Wetland.

In terms of time, there were significant differences in the mercury content of water bodies in the same wetland in different seasons. As can be seen from **Figure 3**, the total mercury concentrations adsorbed by wetlands generally showed a seasonal decrease in all wetlands except Donglai South Street Wetland and Bayhood Wetland. The Donglai South Street Wetland and Bayhood Wetland showed a gradual decrease with seasonal changes in spring, summer and fall, and an increasing trend in winter.

### 3.2 Mercury content levels in the sediment samples

**Figure 4:**
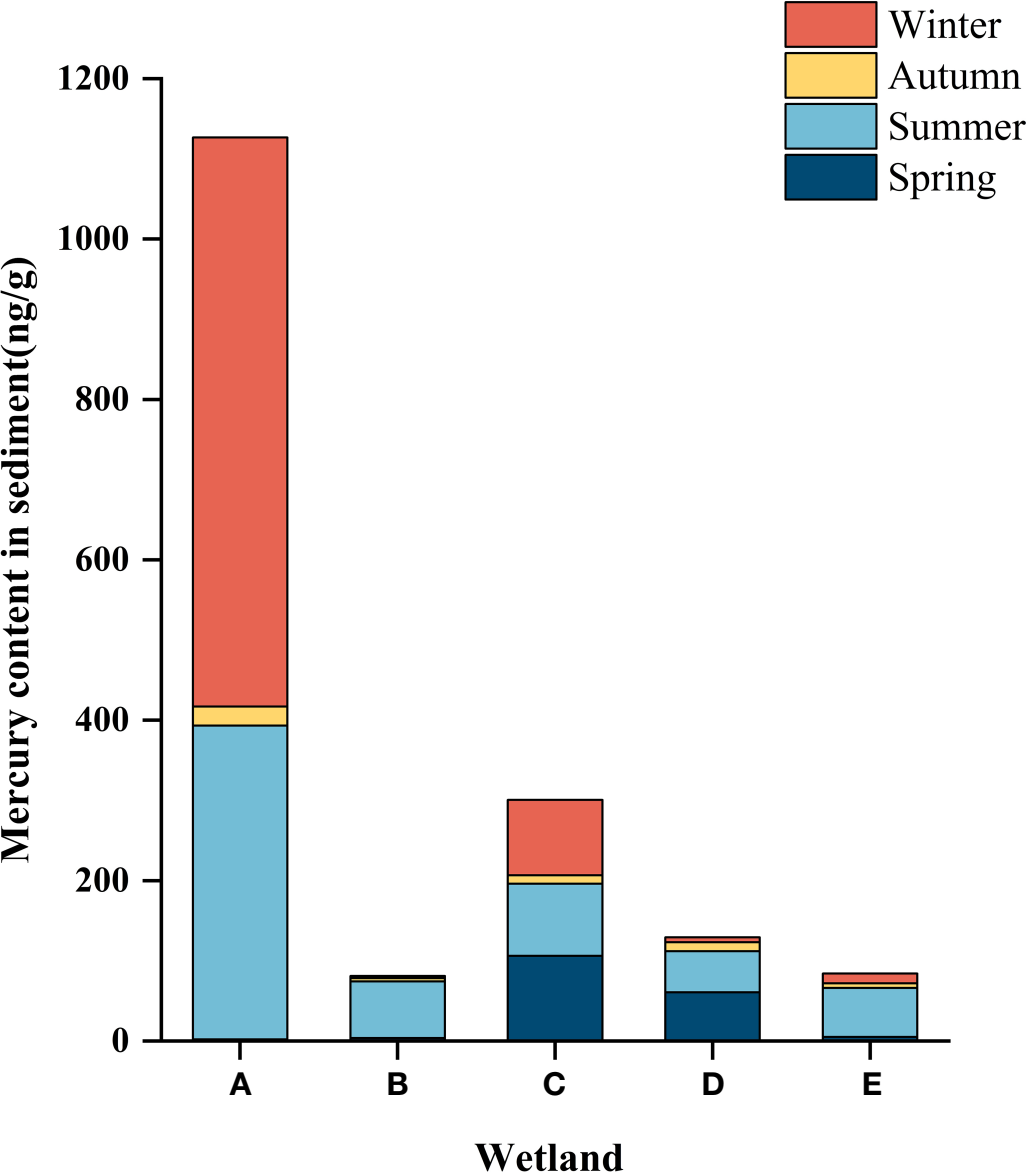
Hg content in the sediment of different constructed wetlands. **(A)** South Fifth Ring Wetland; **(B)** Rival Wetland; **(C)** Beihai Wetland; **(D)** Donglai South Street Wetland; **(E)** Bayhood Wetland.

Sediment layers are the product of gravitational deposition and adsorption. Therefore, the study of sediments can reveal the history and current status of wetland pollution. As shown in **Tables 4** and **5**, the mercury content in sediments of different wetlands in different seasons varied significantly, and the concentration of total mercury in sediments of different wetlands in different seasons ranged from 1.9 ± 0.264 ng/g to 709.33 ± 15.631 ng/g. As can be seen from **Figure 4**, the spatial distribution of the total mercury concentration in the sediments showed a fluctuating tendency along with the flow direction of the river. A gradual downward trend from the South Fifth Ring Wetland in the upper reaches of the river to the Beihai Wetland in the middle reaches of the river where total mercury mass concentrations begin to increase, followed by a downward trend in the lower reaches of the river.

**Table 4:** Description and statistics of the mercury content levels in the sediment of the constructed wetland systems (unit: ng/g).

Wetland	Mercury content in sediment(ng/g)			
	Spring	Summer	Autumn	Winter
South Fifth Ring Wetland	1.9 ± 0.264	391.333 ± 106.226	24 ± 4.582	709.333 ± 15.631
Rival Wetland	3.6 ± 0.200	70.667 ± 4.041	4 ± 1.000	3 ± 1.732
Beihai Wetland	106.3 ± 7.627	90 ± 6.083	10.333 ± 4.509	94 ± 2.646
Donglai South Street Wetland	60.6 ± 3.418	51.333 ± 9.504	11.333 ± 6.658	6 ± 4.583
Bayhood Wetland	5.1 ± 0.529	61 ± 7.000	6 ± 2.646	12 ± 1.000

**Table 5:** Seasonal differences in mercury levels in sediment of different wetlands are significant.

	Spring	Summer	Autumn	Winter	P
South Fifth Ring Wetland	1.80 (1.75,2.00)	399.00(340.00,446.00)	25.00(22.00,26.50)	707.00(701.00,716.50)	0.016
Rival Wetland	3.60 (3.50,3.70)	70.00(68.50,72.50)	4.00(3.50,4.50)	2.00 (2.00,3.50)	0.075
Beihai Wetland	107.90(102.95,110.45)	87.00(86.50,92.00)	10.00(8.00,12.50)	95.00(93.00,95.50)	0.024
Donglai South Street Wetland	61.00(59.00,62.40)	51.00(46.50,56.00)	8.00(7.50,13.50)	5.00 (3.50,8.00)	0.028
Bayhood Wetland	4.90 (4.80,5.30)	58.00(57.00,63.50)	5.00(4.50,7.00)	12.00(11.50,12.50)	0.025
P	0.009	0.013	0.028	0.012	

**Figure 5:**
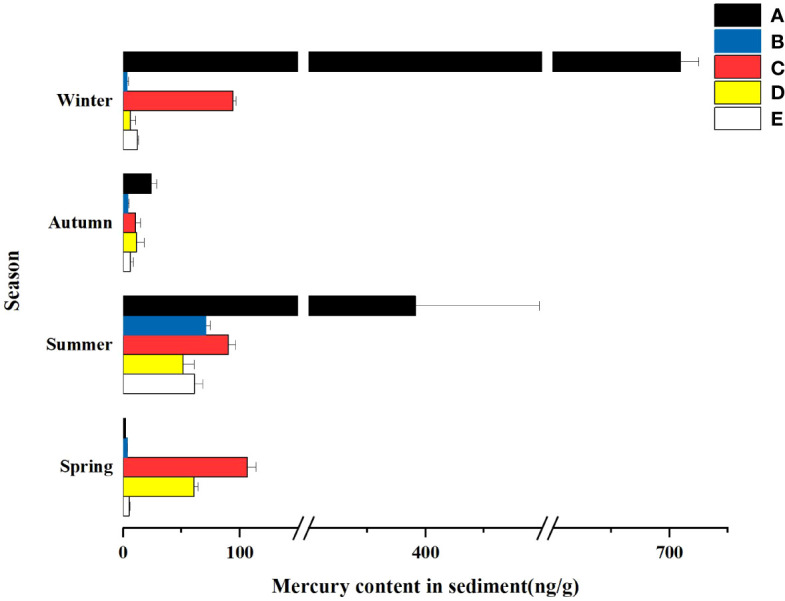
Hg content in the sediment of different constructed wetlands. **(A)** South Fifth Ring Wetland; **(B)** Rival Wetland; **(C)** Beihai Wetland; **(D)** Donglai South Street Wetland; **(E)** Bayhood Wetland.

As can be seen from **Figure 5**, the total mercury content of sediments in different wetlands varied significantly with the seasons, in which the total mercury mass concentration in the summer was higher than that in the other three seasons in all four wetlands except the South Fifth Ring Wetland. The five wetlands as a whole showed that the total mercury content was greater in summer and winter than in spring and fall.

### 3.3 Mercury content levels of the plant samples

The mass concentration of Hg in wetland plants is closely related to wetland geological conditions, landscape layout, pollution history, and anthropogenic activities. As shown in **Tables 6** and **7**, in spring, there were significant differences in the total Hg concentrations in different wetland plants. In terms of spatial distribution, an increasing trend was observed from the South Fifth Ring Wetland along the river flow direction, and a slightly decreasing trend was observed in the Beihai Wetland. As can be seen from the figure, the corresponding plant contents in the five wetland areas did not show any outliers.

**Table 6:** Description statistics of the mercury content levels of the plants in the constructed wetland systems (unit: ng/g).

Wetland	Mercury content in plant(ng/g)			
	Spring	Summer	Autumn	Winter
South Fifth Ring Wetland	1.3 ± 0.1	2.567 ± 1.069	2.867 ± 2.421	1.333 ± 1.155
Rival Wetland	7.8 ± 2.052	3.8 ± 2.211	0.9 ± 0.529	6.333 ± 0.577
Beihai Wetland	14.8 ± 4.951	5.7 ± 1.127	5.667 ± 2.309	5.333 ± 2.082
Donglai South Street Wetland	13.5 ± 1.153	2.667 ± 0.577	5.6 ± 1.442	2.333 ± 1.528
Bayhood Wetland	13.5 ± 1.9	4 ± 1.732	1.667 ± 1.155	3.667 ± 2.082

**Table 7:** Seasonal differences in mercury levels in sediment of different wetlands are significant.

	Spring	Summer	Autumn	Winter	P
South Fifth Ring Wetland	1.30(1.2,1.35)	2.00(1.95,2.90)	3.90(2.00,4.25)	2.00(1.00,2.00)	0.401
Rival Wetland	7.90(6.80,8.85)	3.00(2.5,4.65)	0.70(0.60,1.10)	6.00(6.00,6.50)	0.049
Beihai Wetland	14.90(12.35,17.30)	5.10(5.05,6.05)	7.00(5.00,7.00)	6.00(4.50,6.50)	0.087
Donglai South Street Wetland	13.60(12.95,14.10)	3.00(2.50,3.00)	6.00(5.00,6.40)	2.00(1.50,3.00)	0.028
Bayhood Wetland	13.50(12.55,14.45)	5.00(3.50,5.00)	1.00(1.00,2.00)	3.00(2.50,4.50)	0.047
P	0.032	0.146	0.073	0.052	

**Figure 6:**
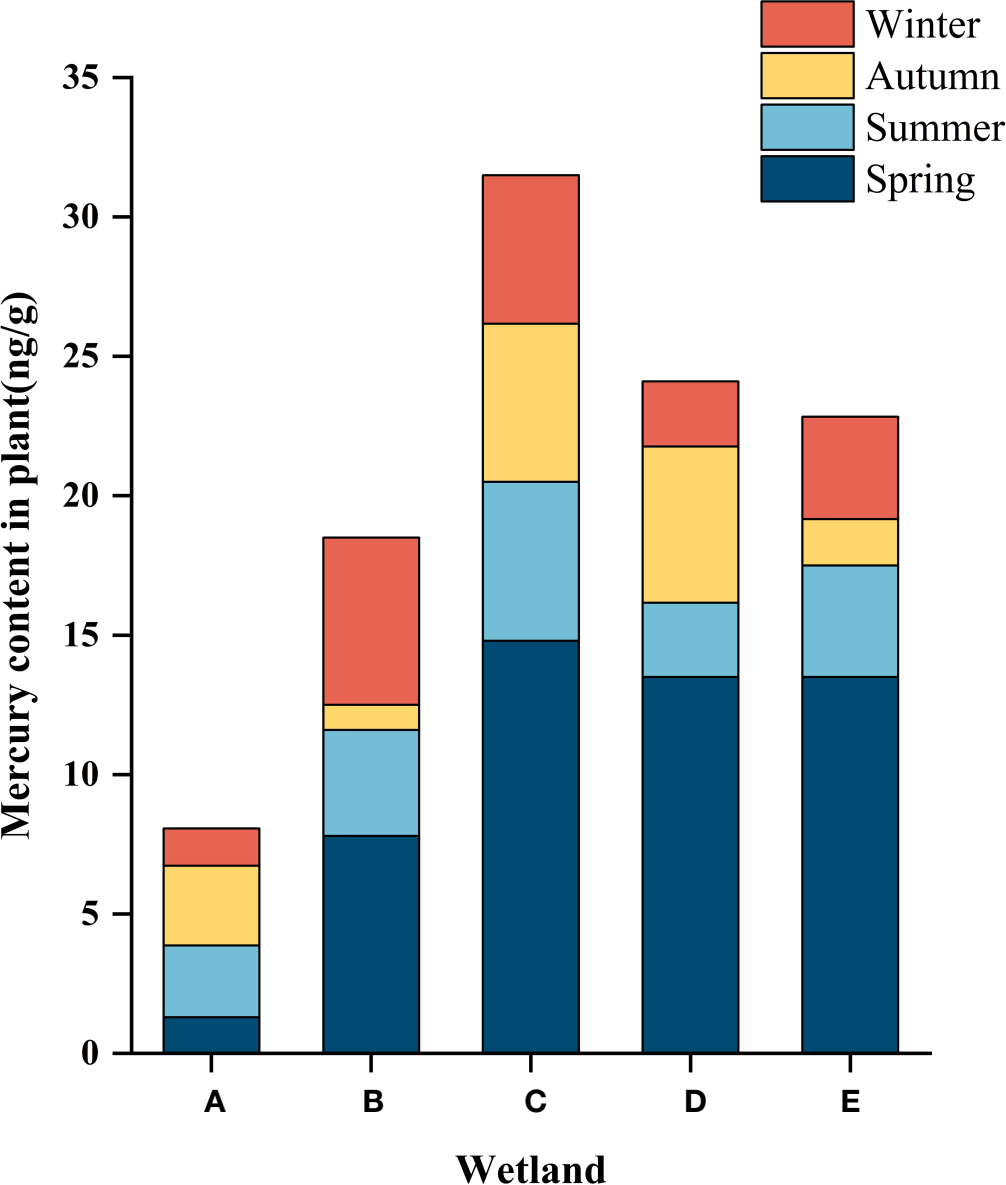
Hg content in the plants of the different constructed wetlands. **(A)** South Fifth Ring Wetland; **(B)** Rival Wetland; **(C)** Beihai Wetland; **(D)** Donglai South Street Wetland; **(E)** Bayhood Wetland.

The characteristics of temporal changes of total mercury in wetland plants are shown in **Figure 6**. Except for the South Fifth Ring Wetland, all other wetlands showed the highest total mercury mass concentration in spring, a slight decrease in summer, and a fluctuating trend of total mercury concentration in the fall and winter seasons, with the Rival Wetland, the Beihai Wetland and the Bayhood Wetland showing a downward and then upward trend, and the other wetlands showing an upward and then a downward trend. However, the total mercury mass concentration generally showed a decreasing and then increasing trend.

**Figure 7:**
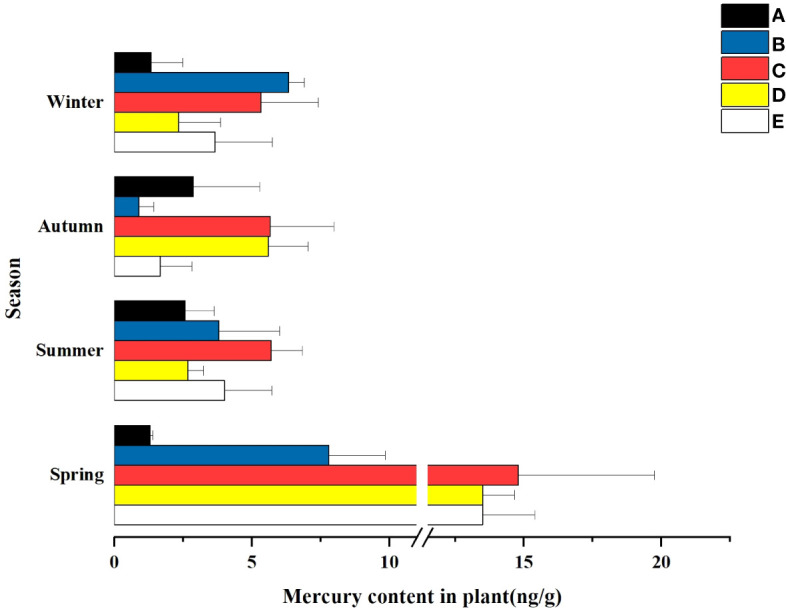
Hg content in the plants of the different constructed wetlands. **(A)** South Fifth Ring Wetland; **(B)** Rival Wetland; **(C)** Beihai Wetland; **(D)** Donglai South Street Wetland; **(E)** Bayhood Wetland.

Temporally, the mass concentration of total mercury in plants in some wetlands had significant differences in different seasons. As can be seen from **Figure 7**, the adsorbed total mercury concentrations in the five wetlands showed a fluctuating trend with seasonal changes. The highest concentration was found in spring, followed by summer and winter, and the lowest total mercury concentration was found in fall. The total Hg concentration in plants peaked at the beginning of the growing season, and with the seasonal change biomass growth rather diluted the mass concentration of Hg in plants, and declined in the maturity period (July-September) and then reached the winter, when the plant uptake stabilized and ceased.

### 3.4 Mercury enrichment in plants

The bioconcentration coefficient of heavy metals reflects the ability of plants to accumulate heavy metals from the surrounding environment (Zhang et al., 2009). Equation 1 was used to calculate the plant mercury enrichment coefficient in wetland. From **Table 8**, it can be seen that the South Fifth Ring wetland plants have the weakest ability to enrich Hg with an enrichment coefficient of 0.0019, and the Bayhood Wetland has the strongest ability to enrich Hg with an enrichment coefficient of 2.6417.

**Table 8:** Biological enrichment coefficients.

Wetland	Spring	Summer	Autumn	Winter
South Fifth Ring Wetland	0.6842	0.0066	0.1194	0.0019
Rival Wetland	2.1667	0.0538	0.2250	2.0000
Beihai Wetland	0.1392	0.0633	0.5484	0.0567
Donglai South Street Wetland	0.2228	0.0519	0.4941	0.3889
Bayhood Wetland	2.6471	0.0656	0.2778	0.3056

### 3.5 Correlation of mercury levels in water, sediment and plants

**Figure 8:**
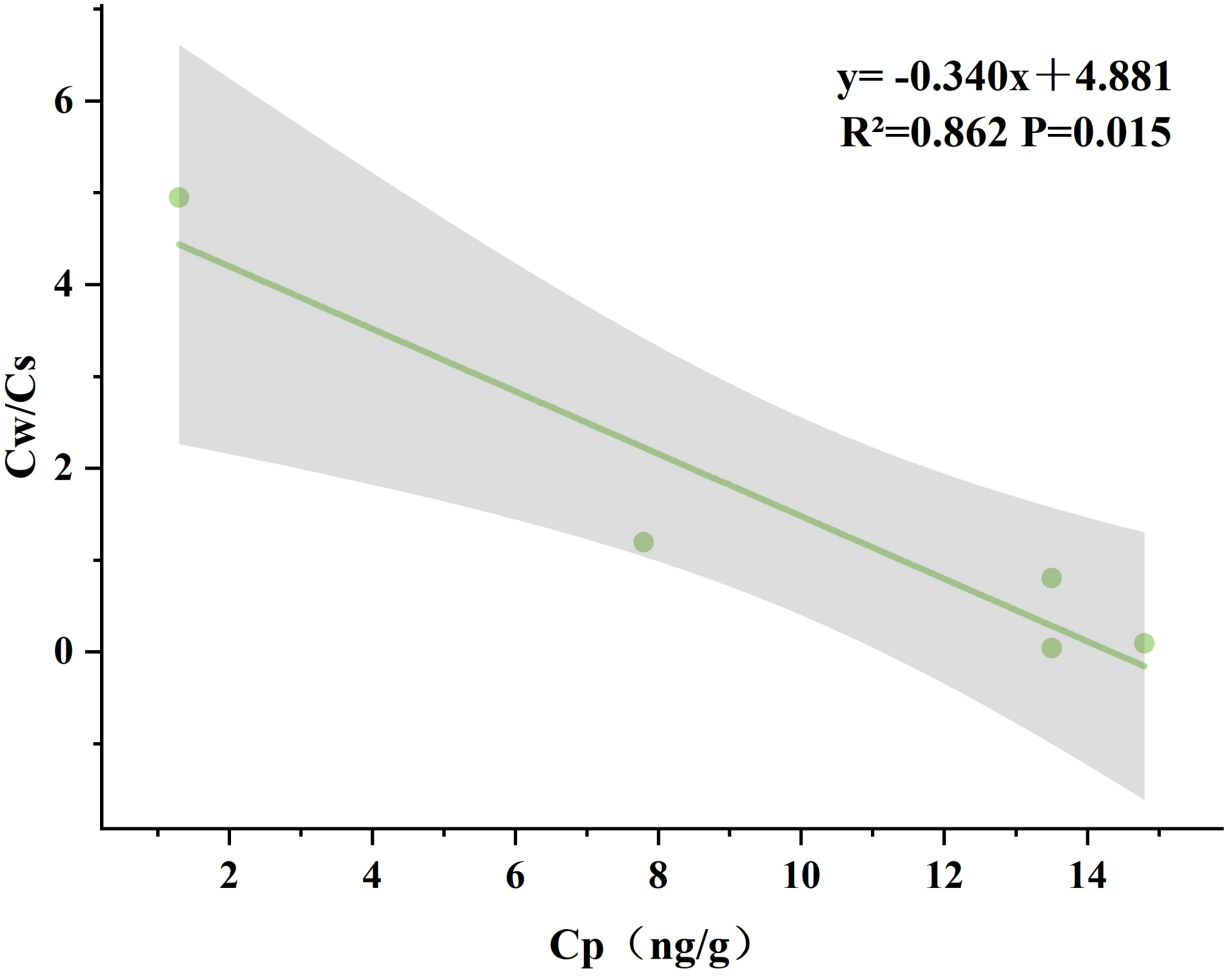
Correlation of mercury concentrations in plants with the ratio of mercury concentrations in water and sediment during springtime.

In this study, the correlation between the mass concentration of Hg in plants (Cp) and the ratio of Hg concentration in water and sediment (Cw/Cs) in different seasons was analyzed simultaneously using Pearson’s test. The results showed that there was a strong correlation between Cp and Cw/Cs only in spring (R²=0.862, P=0.015), as shown in **Figure 8**. No clear correlation was found between Cp and Cw/Cs in the other three seasons. Therefore, the enrichment capacity of plants was strongly influenced by the soil and water environment in spring, and the adsorption capacity of wetland sediments and water bodies for Hg was higher than that of the other three seasons in spring.

## 4 Discussion

### 4.1 Content characteristics of the mercury in the water samples

#### 4.1.1 Characterization of the spatial distribution of mercury in water bodies

Concentration changes within wetlands are susceptible to elevated total mercury concentrations in wetlands that are forced to accept large amounts of foreign water due to the high volume of water coming from upstream and the complexity of water quality changes in flow rates. Rivers flowing through human settlements are susceptible to anthropogenic pollution, and in densely populated areas with well-developed road networks and concentrated industrial facilities, there is a significant increase in human activities, which may be an important factor in the accumulation of mercury (Li et al., 2019b; Prajapati et al., 2022). River input is a major source of mercury into the Yitong River, and the South Fifth Ring Wetland is located at the mouth of the Yitong River, with Xinlicheng Reservoir upstream of it, which opens the gate and injects ecological water into the Yitong River on April 28th every year, resulting in the high mercury content in the water body of the South Fifth Ring Wetland to reach 9.4 ± 0.2 ng/L. Due to the self-purification effect of the urban artificial wetland itself, the mercury concentration in the water body decreased significantly after flowing through the Rival wetland, but due to the diverse sources of pollution in the city, the mercury concentration began to show an increasing trend due to the sinking of foreign mercury sources before and after passing through the Beihai Wetland. The sampling site of Beihai wetland is located near the population gathering area, which has a high content of heavy metal mercury in the water body of the wetland, and in this area, the sources of mercury discharged to the wetland may be mainly domestic sewage, coal combustion, and automobile exhaust (Fulkerson et al., 2007). In addition, it was reported that several culverts in the Changchun section of the Yitong River discharged water into the river, which may also contribute to the elevated Hg content in the wetland.

#### 4.1.2 Characterization of the temporal distribution of mercury in water bodies

The impact of climate change on the Hg cycle is significant, and Hg exposure is likely to increase with regional and globalized warming (Moura and de Lacerda, 2022). Wet and dry deposition is an important pathway for the transfer of heavy metal mercury from pollution sources to the surface of ecosystems. Atmospheric mercury deposition is also one of the major sources of mercury in water bodies and terrestrial ecosystems (Wang and Yan, 2015). The annual average concentration level of total mercury in precipitation in Changchun City, Jilin Province, China, was reported to reach 354 ng/L, and the wet precipitation flux of atmospheric mercury was 152.4 ug/m^2^ (Fang et al., 2004). It was shown that the concentration levels of mercury in atmospheric mercury wet precipitation in the Changbai Mountain region ranged from 11.5 ng/L ~ 18.6 ng/L during the period of 2005 - 2006, with the lowest average total mercury concentration in summer (Wan et al., 2009). During wet atmospheric deposition, surface runoff dissolves Hg into surface soils, resulting in higher levels of Hg in surface runoff than those detected in precipitation (Heng et al., 2011). The annual Hg export rate from surface runoff has been estimated to be 18.24 μg/m^2^ (Gabriel et al., 2011). Natural conditions such as precipitation can exacerbate streambank erosion and alter the distribution of Hg along stream channels (Fornasaro et al., 2022). For example, rainfall can wash away mercury from the catchment leading to a threefold increase in mercury concentration in rivers (Gebka et al., 2018). Temperature, solar radiation and monsoon winds also affect mercury concentration in rivers (Iordache et al., 2022). The Bayhood wetland is located in the urban area of Changchun City, and its shoreline is dominated by cement blocks, except for the artificial wetland itself, which has the ability to purify pollution. Therefore, it is difficult for rainwater to filter out mercury from the soil near the shoreline and enter the wetland water body through the cement block layer. This may explain the low mercury levels in the Bayhood Wetland. Transformation of Hg and release of reactive Hg into the water column can be triggered by solar radiation and increased plankton biomass in the spring, as well as by rapid degradation of organic matter in the fall (Ravichandran, 2004; Saniewska et al., 2010). Resulting in significantly higher levels of mercury in the water column in spring than in the other three seasons is in agreement with the findings of Dominika Saniewska et al (Saniewska et al., 2014).

### 4.2 Content characteristics of the mercury in the sediment samples

#### 4.2.1 Characterization of the spatial distribution of mercury in sediments

Mercury release and adsorption is a two-way process. In this study, it was determined that the high mercury content in the sediments of the South Fifth Ring Wetland may be due to the high mercury content in the water body of the South Fifth Ring Wetland, and the deposition and accumulation of mercury in the sediments by the suspended sediment, which resulted in the high concentration of total mercury in the sediments. Similar to the distribution of mercury in the water body, the concentration of mercury in the sediments showed a decreasing trend from the upstream to the downstream of the river, and an increasing trend before and after the Beihai Wetland in the middle reaches of the river. This section flowed through the urban populated area, indicating the influence of urbanization on the total Hg concentration in sediments. In Shanghai, one of the most prosperous cities in China, the total Hg content in the surface sediments of several lakes showed a clear urbanization pattern, which indicates that heavy metal pollution from anthropogenic emissions accumulates more in the urban center (Li et al., 2013). The high mercury content in the sediments of the Beihai wetland may be due to the fact that the sampling site was located near a sidewalk and was greatly disturbed by tourists and car exhaust, resulting in a relatively high mercury content in the sediments of this wetland. Regarding the Donglai South Street wetland, the high mercury content in the sediments of this wetland may be related to the fact that the sampling site is located east of Donglai South Street, which is the most enriched residential area in the vicinity. Pedestrians and vehicles were frequently seen, causing significant anthropogenic disturbance. In addition, plastic bags, dead branches, and other trash were clearly visible on the water surface, making the wetland a poor environment. In addition, at the bottom of Donglai South Street, an artificial concrete channel was formed during the late construction period. Therefore, the mercury in the wetland sediments may have come not only from the water column and plants, but also from industrial waste left over from the restoration of the channel. Another factor may be related to the fact that the Donglai South Street wetland is a relatively new wetland, and the heavy metal mercury in the sediments cannot be effectively released in a short period of time through exchange between the water body and plants.

#### 4.2.2 Characterization of the temporal distribution of mercury in sediments

Accumulation, diffusion and release of Hg in sediments may be the key factors influencing Hg concentrations in wetlands during dry and wet seasons (Chen et al., 2020). According to Fick’s law, TMeHg and THg levels in sediments are higher in the wet season than in the dry season. Both hydrodynamic perturbations and higher diffusion rates can lead to increased Hg release. In addition, the concentration of precipitation in the rainy season may result in land-based sources of pollutants being flushed to wetlands through surface runoff and rivers (Dong et al., 2015), and the average sediment content in the rainy season is greater than that in the dry season due to the large amount of sediment brought into the wetlands by rivers and surface runoff, leading to increased Hg content in wetland sediments. Moreover, increased hydrodynamic activity in the rainy season disturbs the lake bottom sediments, leading to an increase in the release of Hg from the sediments, which is consistent with the findings of Wang et al (Wang et al., 2019). In addition, another reason for higher Hg levels in summer at each sampling site was due to higher water temperatures in summer than in the other three seasons. It was found that higher water temperatures favored the release of sediment Hg into the water column, which is consistent with the findings of Liu Weihao et al (Fan et al., 2019).

### 4.3 Content characteristics of the mercury in the plant samples

#### 4.3.1 Characterization of the spatial distribution of mercury in plants

Phytoremediation is an effective and environmentally friendly method for cleaning contaminated soil, natural ponds and wastewater (Chang et al., 2022). Some natural macrophytes have a great potential to accumulate metals in their bodies, helping to remove them from various sources (Mishra and Tripathi, 2008). A study found that the mercury concentration in mangrove wetland plants in China was about 10.2 ± 6.8 ng/g (Ding et al., 2010), which is in the same order of magnitude as the total mercury content levels in national and international research results, but the magnitude is different. In this study, the concentration of mercury in plants showed a fluctuating trend with the river, starting from the South Fifth Ring Wetland the concentration of mercury in plants gradually showed an increasing trend to the Beihai Wetland where it reached a peak of 14.8 ± 7.0 ng/g and then began to decline. The highest level of Hg in plants in the Beihai wetland may be attributed to the small size of the wetland, poor water mobility, and the relatively closed wetland environment, as well as the obvious urbanization near the Beihai wetland, which is susceptible to human disturbances including the influx of domestic waste, industrial waste pollution, and other Hg-containing pollutants. Compared with the range of Hg concentrations in plant species from 0.14 mg/kg to 81.56 mg/kg in a test58 in Spain (Fernández-Martínez and Rucandio, 2013; Fernández-Martínez et al., 2015), the total Hg mass concentrations in plants in this study were low. Although the total Hg mass concentrations in plants were lower than in other parts of the world, the presence of Hg in this region still follows a gradient of water flow.

#### 4.3.2 Characterization of the temporal distribution of mercury in plants

Hg concentrations in plants were significantly higher in spring than in the other three seasons, and comparable in the other three seasons. Hg concentrations in plants peaked at the beginning of the growing season, when plant biomass was low and the ability to absorb and enrich Hg was high, resulting in higher Hg levels in plants. In summer, when the temperature is favorable and the metabolic activity of plants is strong, the roots and leaves of plants are more capable of absorbing Hg from the environment, and at the same time, with the growth of plant biomass, the Hg in plants is diluted, so the concentration of Hg in plants in summer is lower than that in spring. In addition, abundant rainfall and high-water flow in summer result in dilution of mercury in water bodies, so the concentration of mercury in plants is lower. In the fall, plant biomass stabilizes and gradually stops growing, and the ability of plants to enrich with mercury gradually decreases. In winter, the temperature decreases, plant growth stops, and some wetlands harvest plants to achieve the effect of mercury purification, so the concentration of mercury in plants in winter is comparable to that in fall.

### 4.4 Characterization of mercury enrichment in plants

The higher the plant enrichment factor, the higher the uptake capacity of wetland plants for certain heavy metals. However, it also reflects the stronger ability of heavy metal elements to migrate from water and soil to plants. In this study, it was found that the enrichment coefficients of Hg in plants of five artificial wetlands generally showed a decreasing trend along the river flow to the Bayhood Wetland and began to increase. This may be related to the pollution load to which the artificial wetlands are subjected, with higher pollution loads in the upper reaches of the river and higher Hg content in the wetlands, so the plants would adsorb more Hg. To the lower reaches of the river, the downstream pollution load is lower due to the reduction effect, so the amount of mercury adsorbed by plants is limited. As can be seen from the table, the enrichment coefficient BCF of plants in Rival Wetland and Bayhood Wetland was > 1. This indicated that the plants in these two wetlands had a strong capacity to enrich Hg and a strong resistance to Hg. However, the enrichment coefficients of the plants in these two wetlands were still far below the critical criterion for an ultra-enricher, and therefore still not within the range of an ultra-enricher (Mishra and Tripathi, 2008). During the spring sampling in this study, wetland plants in the Rival Wetland were removed and harvested, and only the below-ground portions of the plants were collected for sampling. The below-ground portion of plants is the main source of Hg enrichment and storage. The mercury content of aboveground plant tissues is only 1/198 of the mercury content of underground tissues, while the mercury content of underground plant tissues may be more than three times of the mercury content of the sediments around the plant’s root living environment (Mishra and Tripathi, 2008). Therefore, the enrichment factor of plants in the Rival Wetland in the spring is highly contingent.

### 4.5 Correlation of mercury levels in water, sediment and plants

As shown in **Figure 8**, mercury concentration levels in sediments and water bodies began to decrease gradually with the increase of mercury content in plants at the beginning of spring growth. This phenomenon can be attributed to the biosorption of Hg by the plants and the uptake and transport mechanism of Hg by the root system (Patra and Sharma, 2000). The enrichment of Hg within the plant gradually increased with further growth of the plant. However, when the Hg content in plants reaches a certain saturation value, the Hg content in sediments and water bodies will no longer show a significant decreasing trend. This may be because the rate of uptake of Hg by plants is in equilibrium with the rate of its accumulation and release. Therefore, it is believed that regular harvesting of aboveground plant parts plays an important role in the growth and reproduction of plants and the purification effect of artificial wetlands. In summary, proper harvesting of aboveground plants can promote the activities of microorganisms in artificial wetlands and the absorption and enrichment of nutrients. In addition, effective harvesting measures can remove the nutrients absorbed by plants and prevent the release of pollutants caused by plant shedding and decay, thus improving the removal effect of pollutants from artificial wetlands (Verhofstad et al., 2017).

### 4.6 Potential ecological risks

From 2012 to 2017, the changes in the demand industry structure and demand category structure led to large increases in atmospheric mercury emissions from the cement, lime, and gypsum processing industries. Meanwhile, the changes in the production structure led to large increases in atmospheric mercury emissions from the non-ferrous metal smelting and rolling processing industries (Wu et al., 2021). Atmospheric subsidence, surface runoff, and mercury brought out of riverbank soil into river bodies through leaching are considered to be the main sources of mercury content in riverbed mud. The bottom soil can provide nutrient-rich soil fertilizer and promote plant growth. According to the findings of some previous studies, the content of several heavy metals in the river sediment in a certain area was ranked from high to low. It was found that the content levels of heavy metal mercury were the lowest. However, the potential ecological risk assessment results showed that mercury was the highest among the detected several heavy metals, and should be considered as a medium to high ecological risk. Therefore, attention should be paid to the ecological risks of mercury pollution during river dredging and sediment land utilization processes (Lu et al., 2018).

### 4.7 Circulation of total mercury in urban constructed wetlands

**Figure 9:**
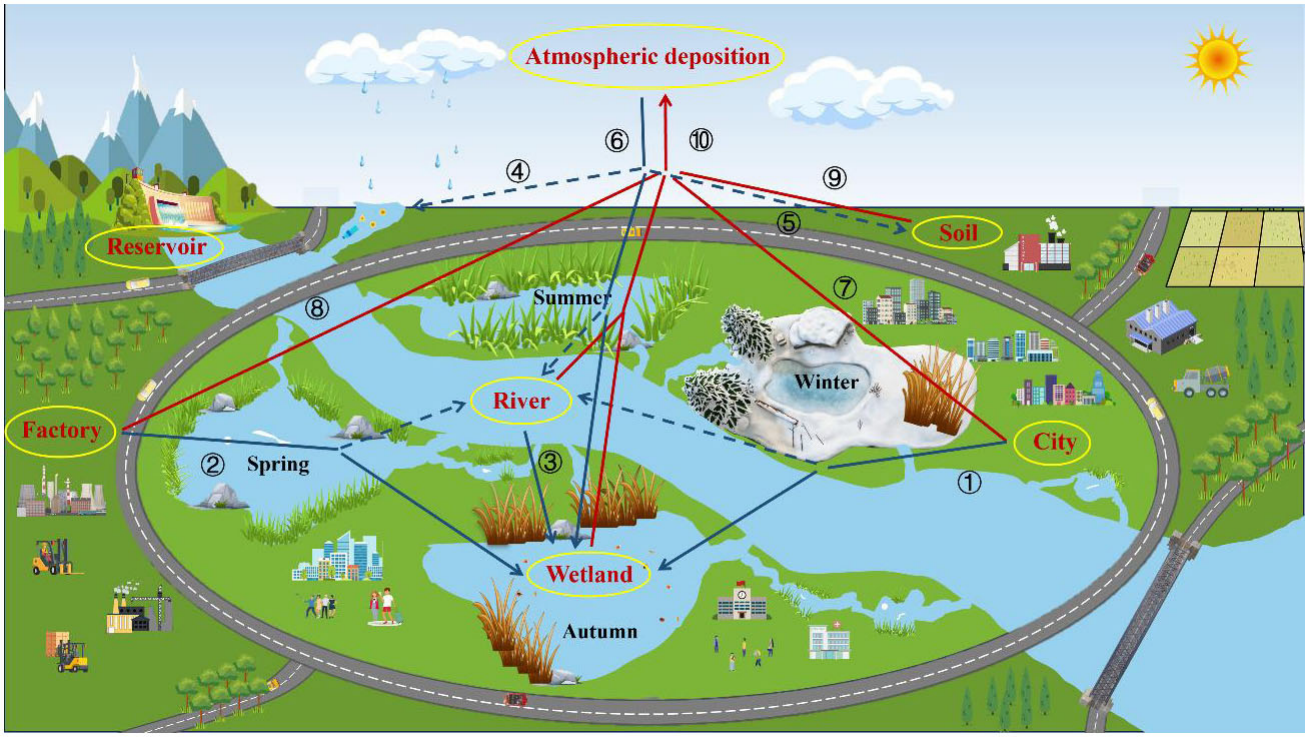
Circulation of total mercury in urban constructed wetlands. (The thick blue arrows are the main the pathway by which Hg sinks from other Hg reservoirs into wetland Hg reservoirs; The thick red arrows are the main the pathway of mercury from other mercury reservoirs to the atmospheric mercury reservoirs. The dashed lines represent indirect mercury banking pathways for mercury. ①:Domestic wastewater ②:Industrial wastewater ③ :River input ④⑤:Surface runoff ⑥:Atmospheric deposition ⑦:Man induced activities (automobile exhaust, etc.) ⑧:Industrial gas emissions ⑨:Evaporation from the soil ⑩:Evaporation from the water body.).

From **Figure 9**, it can be seen that mercury generated through river input, surface runoff, sewage discharge, etc. will enter water bodies and thus sink into artificial wetlands. The mercury generated through water, soil evaporation, factory emission, human activities, etc. enters into the atmosphere, and then part of it directly falls into the wetland through atmospheric deposition, part of it indirectly sinks into the wetland through surface runoff, and the mercury that sinks into the wetland ultimately returns to the atmosphere through evaporation, thus forming the cycle of mercury in the urban artificial wetland. In this cycle, the plants growing in the artificial wetland can absorb the enriched mercury and through the unique management of the artificial wetland, the plants in the wetland can be harvested in the winter to effectively reduce the load of mercury in the city, and ultimately to maintain the stability of the mercury in the urban environment and avoid the risk of overexposure.

## 5 Conclusions

In this study, we selected five wetlands in the Changchun section of the Yitong River and measured the total mercury content in the water, sediments and plants in each wetland. We observed that the mercury content in the water bodies, sediments and plants of the five wetlands showed a fluctuating trend with the river flow direction and had certain spatial and temporal distribution characteristics. The factors affecting the spatial distribution of total Hg in the wetlands may be the geological conditions of the wetlands, the history of pollution, anthropogenic activities, and the landscape layout. Factors affecting the temporal distribution of total mercury in wetlands may be temperature, rainfall, etc. The mercury content in plants varies greatly with the growing season, which is mainly related to the growth characteristics of plants. Comparison of the correlation between the Hg content in plants and the ratio of water and sand concentration in different seasons revealed that the level of Hg content in plants in spring had a strong correlation with the ratio of water and sand concentration (R²=0.862; P<0.05), indicating that the adsorption of Hg by plants was stronger in the early growth period. Among the bioconcentration factors, the BCF of the Beihai wetland in spring and winter and the Bayhood wetland in spring were all greater than 1, indicating that the plants in these two wetlands had a stronger ability to adsorb enriched Hg, and the plants in the urban man-made wetland also had a certain ability to enrich Hg, which could alleviate the Hg pollution of the water body to a certain extent. Overall, the wetland ecosystem showed a reduction trend in total Hg output from the downstream watershed. This may be due to the retention of particulate matter by the aquatic plants in the artificial wetland as well as the sedimentation effect produced by the long-time hydraulic retention and the regular salvage of aquatic plant litter. At the same time, urbanization and industrialization affect mercury levels in aquatic environments, so that the risk of residential exposure needs to be looked at.

## Data availability statement

The original contributions presented in the study are included in the article/supplementary material. Further inquiries can be directed to the corresponding authors.

## Author contributions

JZ: Conceptualization, Data curation, Methodology, Writing – original draft, Writing – review & editing. HZ: Data curation, Investigation, Software, Validation, Writing – review & editing. XL: Software, Visualization, Writing – review & editing. XB: Formal analysis, Investigation, Writing – review & editing. YH: Data curation, Visualization, Writing – review & editing. DC: Funding acquisition, Project administration, Writing – review & editing. ZW: Funding acquisition, Project administration, Resources, Supervision, Writing – review & editing. GZ: Conceptualization, Formal analysis, Funding acquisition, Methodology, Project administration, Resources, Writing – review & editing.
